# Unraveling a Unique Encounter of Fusobacterium nucleatum With Empyema: A Case Report and Review of Literature

**DOI:** 10.7759/cureus.42488

**Published:** 2023-07-26

**Authors:** Raghavendra R Sanivarapu, Ramya Sruthi Rajamreddy, Ateet Kosaraju

**Affiliations:** 1 Pulmonary and Critical Care Medicine, Texas Tech University Health Sciences Center, Midland-Odessa, USA; 2 Pulmonary and Critical Care Medicine, Nassau University Medical Center, East Meadow, USA; 3 Internal Medicine, People's Education Society Institute of Medical Sciences and Research (PESIMSR), Kuppam, IND; 4 Internal Medicine, Nassau University Medical Center, East Meadow, USA

**Keywords:** complex pleural effusion, complicated community-acquired pneumonia (ccap), fusobacterium nucelatum, loculated pleural effusion, pulmonary empyema

## Abstract

We report a case of *Fusobacterium nucleatum* (*F. nucleatum*)* *empyema in a 34-year-old male with no significant past medical history or obvious risk factors who presented with shortness of breath and chest pain. His imaging showed complicated parapneumonic effusion which grew *F. nucleatum. *He was started on piperacillin-tazobactam. The patient's clinical condition deteriorated despite initial therapeutic efforts, leading to escalated antibiotic therapy and further investigations. The patient's subsequent clinical course included pigtail catheter placement with drainage of fluid requiring tpa and dornase alpha, leading to significant improvement and eventual discharge on oral amoxicillin-clavulanic acid.

## Introduction

Empyema, the accumulation of pus within the pleural space, is a severe complication of bacterial pneumonia and can lead to significant morbidity and mortality. The most common causative organisms are *Streptococcus pneumoniae* and *Staphylococcus aureus. Fusobacterium nucleatum *(*F. nucleatum*), an anaerobic gram-negative bacillus traditionally associated with oral and gastrointestinal diseases, has recently been implicated in various severe invasive infections, including empyema, a pus-filled infection in the pleural space [1,2]. While *F. nucleatum* infections are rare, their presentation is often atypical and associated with substantial morbidity and mortality [3]. The common infection caused by these bacteria include oral and periodontal infections. In recent years, emerging case reports have highlighted the need to consider *F. nucleatum* in the differential diagnosis of culture-negative empyemas, particularly when the standard empiric antibiotic therapy fails [4,5]. Here, we present a case of a young male with no risk factors who presented with complicated parapneumonic effusion and pleural fluid cultures grew *F. nucleatum* empyema.

## Case presentation

A 34-year-old male with no past medical history presented with shortness of breath at rest for three weeks. His shortness of breath was associated with productive cough and chest pain and he went to urgent care a week prior to admission to hospital, where he was given pain medications which did not help his symptoms. He smoked cigarettes about a half pack per day and drinks alcohol occasionally. He denied using illicit drugs. He worked in an oil factory and had exposure to dust, mold, and fumes for about three years.

**Figure 1:**
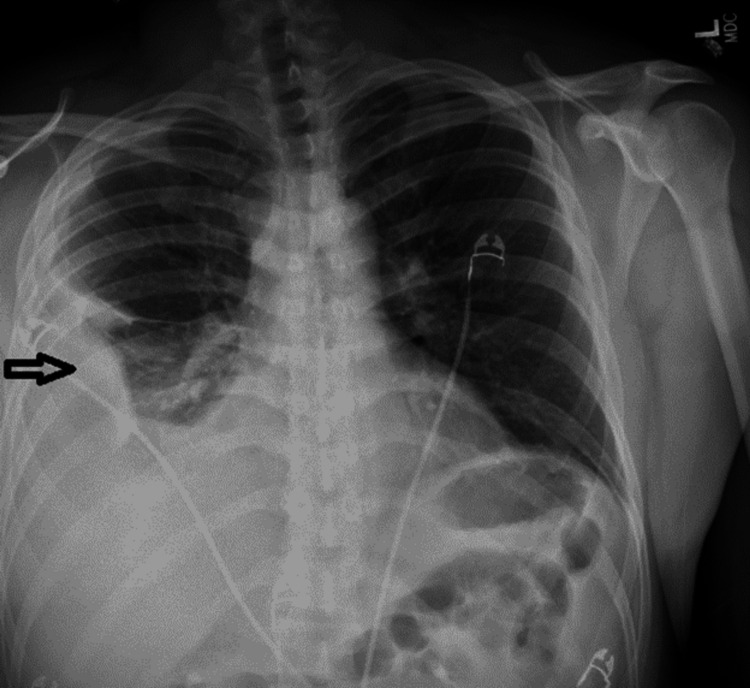
Admission chest X-ray showing a right-sided pleural effusion (arrow)

**Figure 2:**
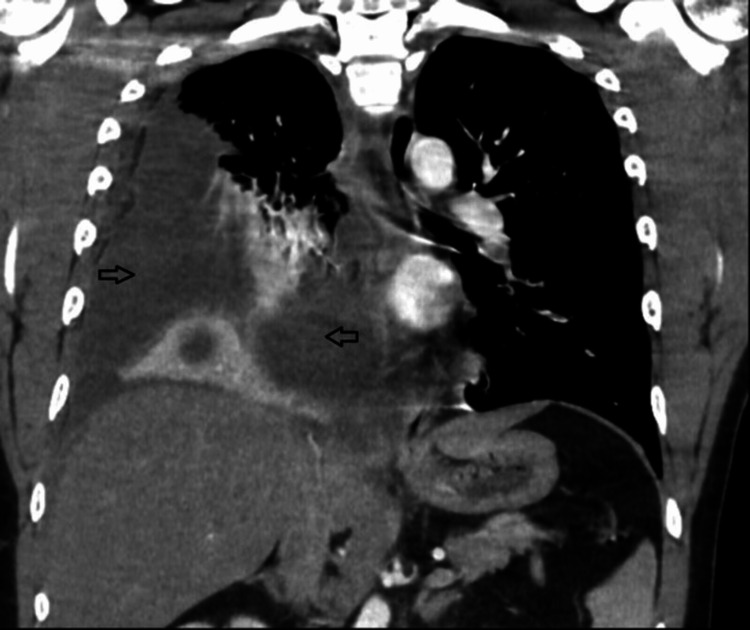
CT scan of the chest coronal view showing loculated pleural effusion (arrows) on admission

On admission, his vitals are the following: blood pressure: 123/69 mmHg; heart rate: 130 bpm; and temperature: 102.5 F. His physical exam showed reduced breath sounds on the right side with dullness to percussion. He was tachypneic, tachycardic, and distressed from pain. His dentition was normal and no caries or cavities were noted on the exam. The rest of the exam was normal. He was given intravenous normal saline continuously 100 cc/hr and morphine 5 mg for pain. His lab analysis was significant for leukocytosis 20.8X 10*3/uL and elevated procalcitonin at 4.24 ng/mL. The rest of the labs including basic metabolic panel, liver-related tests, and urine analysis were normal. A chest X-ray on admission showed right-sided pleural effusion with atelectasis, as shown in Figure 1. CT of the chest showed a loculated right-sided large pleural effusion with associated atelectasis and consolidation as shown in Figure 2.

The patient was started on 4.5 grams of intravenous piperacillin-tazobactam and had a thoracentesis done in the emergency department for severe shortness of breath with the removal of 1.5 L of purulent pleural fluid. The repeat chest X-ray post thoracentesis showed persistent moderate effusion despite the patient's improved symptoms. Given his persistent effusion and leucocytosis, his antibiotics were escalated to linezolid and meropenem.

**Figure 3:**
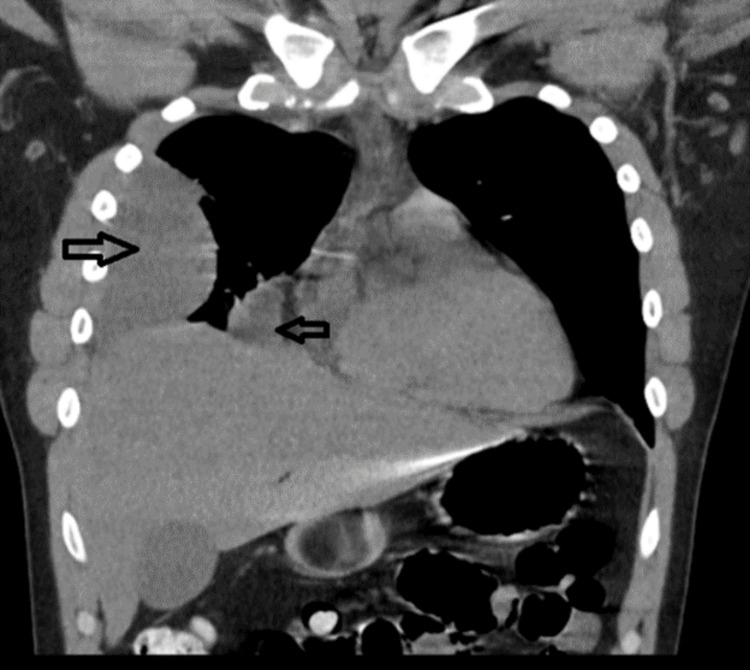
Repeat CT scan of chest showing reduced pleural effusion with anteriorly located loculated effusion (arrows)

A repeat CT scan of the chest was done on day two and showed an anterior loculated effusion, as shown in Figure 3. A pigtail catheter was placed with draining of yellow purulent fluid. He was given tpa and dornase alpha via chest tube to clear the loculations for a total of six doses over three days, and he drained a total of 3 L of yellow straw-colored pleural fluid.

The pleural fluid analysis showed Ph 6.87 (normal range: 7.25-7.45), pleural fluid glucose <20 mg/dl, and pleural fluid protein 5 g/dl consistent with exudative effusion by light's criteria. The pleural fluid cytology showed acute inflammatory cells and no malignancy. The fluid culture grew *F. nucleatum *which was pan-sensitive. His sputum culture did not show any organism. The other tests are summarized in Table 1. The antibiotics were deescalated to meropenem alone, and his leukocytosis trended down to 8x10*3/uL, procalcitonin dropped to 0.8 ng/ml, and he became afebrile.

**Table 1:** Diagnostic tests performed to find the possible etiology Ag: antigen, Ab: antibody, WBC: white blood cell, RBC: red blood cell

TEST	RESULT
Aspergillus galactomannan Ag	Negative
Cryptococcus Ag	Negative
Histoplasma galactomannan Ag	Negative
Coccidioides immitis Ab	Negative
Pleural fluid WBC	8418 /ul
Pleural fluid RBC	9000/ ul
Blood cultures	Negative

**Figure 4:**
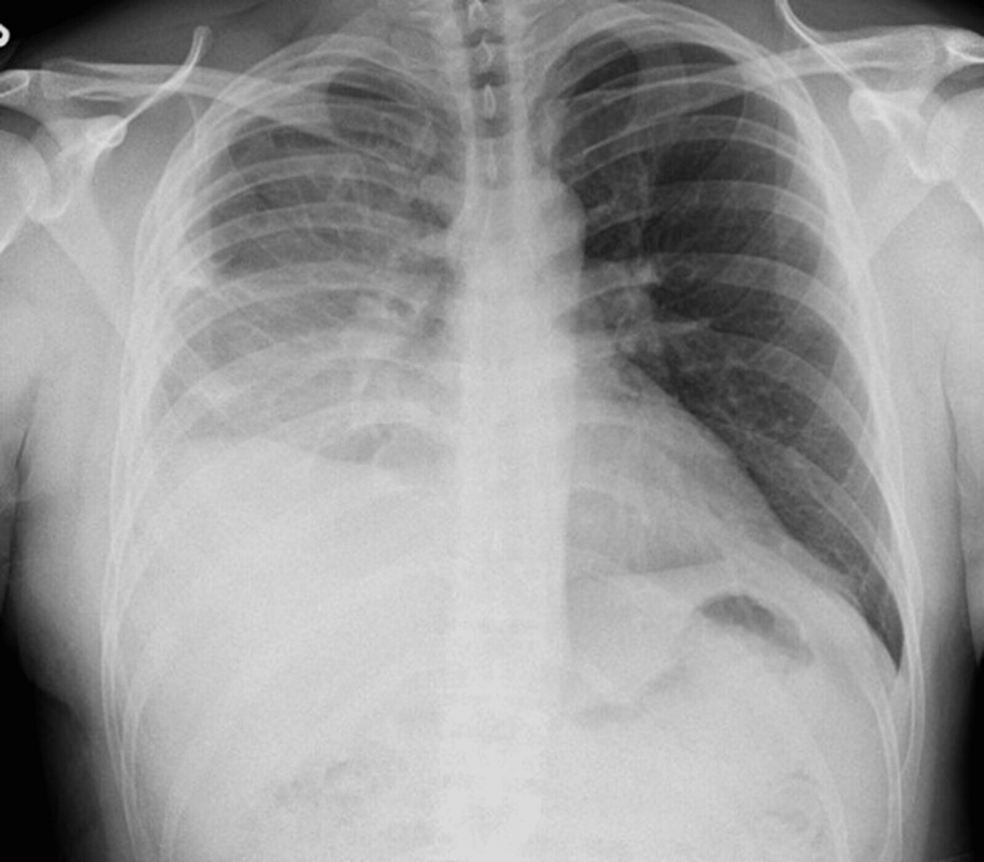
Chest X-ray showing improved aeration of the lung after pig tail catheter removal

The empyema was drained completely, and the chest tube output dropped to <10 ml/day. A repeat chest X-ray showed improved lung aeration with atelectasis in the right lower lobe, as shown in Figure 4. The chest tube drain was removed eventually, and the patient was discharged home after two weeks of hospitalization, and he was given amoxicillin-clavulanic acid for seven days to finish a total of 21 days.

## Discussion

*F. nucleatum*, a common oral commensal and traditionally associated with oral and gastrointestinal diseases, has been recently known to be associated with other invasive infections. Our understanding of *F. nucleatum* empyema has evolved considerably recently. Multiple case reports illustrate that patients with *F. nucleatum* empyema often present with non-specific clinical symptoms, making a definitive diagnosis challenging. The clinical diagnosis is often further complicated by the bacterium's fastidious nature and the limitations of conventional culture techniques [4,6]. Molecular diagnostic methods, such as 16S rRNA sequencing, can help to overcome these challenges and have been instrumental in confirming the diagnosis in numerous cases [2,4].

In recent literature, *F. nucleatum* has been increasingly associated with immunocompromised states, such as rheumatoid arthritis and other underlying co-morbidities [5,7]. This raises the question of whether immunosuppression might predispose individuals to *F. nucleatum* empyema, necessitating further research. *F. nucleatum*'s pathogenic potential is also demonstrated by its association with various forms of empyema, such as pericardial empyema, pleural empyema, and concurrent infections like pyogenic liver abscesses and lung abscesses [6-8]. This pathogen can even extend its infection to skeletal structures, as reported by concurrent spondylodiscitis and osteomyelitis [9,10]. The factors that could have favored infection in our patient could be his occupational exposure and the possibility that he may have had an aspiration event when he had alcohol, and it is only speculation that cannot be definitively proved otherwise.

The role of *F. nucleatum* in severe pleural infections has yet to be fully understood. However, co-infection with other bacteria, such as *Streptococcus intermedius*, might lead to a distinct clinical entity of pleural infections [8]. Furthermore, intriguing correlations have been noted between *F. nucleatum* empyema and malignancies, particularly lung squamous cell carcinoma, which may have important implications for disease prognosis and patient management [11].

The treatment of *F. nucleatum* empyema involves both medical and surgical approaches. As *F. nucleatum* is an anaerobic bacterium, antimicrobial agents effective against anaerobes, such as metronidazole, β-lactam/β-lactamase inhibitor combinations, carbapenems, or clindamycin, are commonly used [12]. High-dose penicillin has also been reported as an effective treatment. However, due to the increasing incidence of antimicrobial resistance, susceptibility testing is recommended to guide appropriate antibiotic therapy [13]. Surgical intervention, including thoracentesis, tube thoracostomy, or even video-assisted thoracoscopic surgery, may be required to evacuate the empyema. The optimal duration of antibiotic therapy for *F. nucleatum* empyema remains undetermined and depends on the individual patient's clinical response, the extent of infection, and whether surgical intervention is involved. However, as a general guideline, it is recommended that antibiotic treatment for pleural infections, such as empyema, be continued for four to six weeks [14]. Extended therapy may be warranted in more severe or complicated cases or in immunocompromised patients. Regular clinical assessment and follow-up imaging should guide the duration of therapy, with the goal of complete resolution of infection. Despite aggressive therapy, *F. nucleatum* empyema carries a significant risk of morbidity and mortality, emphasizing the importance of early recognition and timely initiation of treatment [15]. Table 2 summarizes the reported cases of *F. nucleatum* causing empyema and summarizes the findings.

**Table 2:** A literature review of cases of F. nucleatum-induced pulmonary empyema

Author	Year	Age/Sex	Type of Infection	Clinical Presentation	Diagnostic	Treatment	Associated conditions	Outcome
Lucia et al. [3]	2020	40/M	Pleural empyema	Weight loss, anorexia, night sweats	Pleural fluid cultures	Amoxicillin-clavulanic acid	Poor oral hygiene	Recovered
Park et al. [4]	2023	NA	Brain abscess and pleural empyema	NA	16s rRNA sequence	NA	NA	Recovered
Tang et al. [5]	2021	47/M	Pleural empyema	Chest pain, shortness of breath	Pleural fluid culture	Amoxicillin-clavulanic acid	Rheumatoid arthritis	Recovered
Sun et al. [11]	2023	49/M	Pleural empyema	Cough, fever, chest pain	Pleural fluid culture	Cefoperazone sodium/sulbactam sodium	Squamous cell carcinoma of the lung	Recovered
Gohar et al. [6]	2019	54/M	Liver abscess and pleural empyema	Chest pain, fever, dyspnea, weight loss	Liver abscess cultures and pleural fluid cultures	Metronidazole	NA	Recovered
Reisinger et al. [7]	2019	52/M	Pericardial empyema	Dyspnea, septic shock	Pericardial fluid cultures	Piperacillin/tazobactam & Linezolid	Dental carries, IV drug abuse	Recovered
Bonnesen et al. [9]	2021	77/F	Pleural empyema and spondylodiscitis	Cough, back pain	Pleural fluid cultures and blood cultures	Amoxicillin-clavulanic acid	Smoker	Recovered
Hockensmith et al. [1]	1999	57/M	Pleural empyema	Dyspnea, cough, chest pain	Pleural fluid cultures	Levofloxacin and metronidazole	Smoker	Recovered
Nagaoka et al. [2]	2017	47/M	Pleural empyema	Chest pain, cough	16s rRNA sequence	NA	NA	NA
		60/M	Pleural empyema, cavitary lung lesion	Dyspnea, fever	16s rRNA sequence	NA	NA	NA
Waqas et al. [10]	2018	51/M	Pleural empyema and osteomyelitis	Fever, weight loss	16s rRNA sequence	Clindamycin	Dental caries	Recovered

## Conclusions

This growing body of literature underscores the importance of including *F. nucleatum* in the differential diagnosis of culture-negative empyema, especially in immunocompromised individuals and those with non-resolving symptoms under empirical therapy. Continued research will be crucial in further understanding the pathogenesis, risk factors, and optimal management strategies for *F. nucleatum* empyema.
